# Supplementary material for: Heat adaptation of phage T7 under an extended genetic code
Source: Virus Evol. 2021 Dec 1;7(2):veab100. doi: 10.1093/ve/veab100 (PMC8923235; doi:10.1093/ve/veab100)
Supplement: veab100_Supp [file veab100_supp.zip › Sequencing_Analysis.zip!/Sequencing_Analysis/S3/evidence/SNP_16.html]

BRESEQ :: Evidence


| Predicted mutation | | | | | | |
| --- | --- | --- | --- | --- | --- | --- |
| evidence | seq id | position | mutation | annotation | gene | description |
| RA | NC\_001604.1 | 37,346 | T→C | intergenic (–/–) | – / – | –/– |

| Read alignment evidence... | | | | | | | | | | | |
| --- | --- | --- | --- | --- | --- | --- | --- | --- | --- | --- | --- |
|  | seq id | position | | ref | new | freq | score (cons/poly) | reads | annotation | genes | product |
| \* | NC\_001604.1 | 37,346 | 0 | T | C | 99.1% | 5597.8 / NA | 1867 | intergenic (–/–) | *–/–* | –/– |
| Reads supporting (aligned to +/- strand):  **ref** base T (7/2);  **major** base C (876/961);  **minor** base A (6/10);  **total** (891/976) | | | | | | | | | | | |
| Fisher's exact test for biased strand distribution *p*-value = 4.61e-01 | | | | | | | | | | | |
| Kolmogorov-Smirnov test that lower quality scores support variant *p*-value = 1.00e+00 | | | | | | | | | | | |

Only 100 of 1872 total aligned reads displayed.  

`AGAGTTGAGGCTGCGAAGAGCACTCAAAGAGCAATCGATGCGGTATCTGCTAAGTATCAAGAAGACCTTGCCGCGCTGGAAGGGAGCACTGATAGGATTATTTCTGATTTGCGTAGCGACAATAAGCGGTTGCGCGTCAGAGTCAAAACTACCGGAACCTCCGATGGTCAGTGTGGATTCGAGCCTGATGGTCGAGCCGAACTTGACGACCGAGATGCTAAACGTATTCTCGCAGTGACCCAGAAGGGTGACGCATGGATTCGTGCGTTACAGGATACTATTCGTGAACTGCAACGTAAGTAGGAAATCAAGTAAGGAGGCAATGTGTCTACTCAATCCAATCGTAATGCGCTCGTAGTGGCGCAACTGAAAGGAGACTTCGTGGCGTTCCTATTCGTCTTATGGAAGGCGCTAAACCTACCGGTGCCCACTAAGTGTCAGATTGACATGGCTAAGGTGCTGGCGAATGGAGACAACAAGAAGTTCATCTTACAGGCTTTCCGTGGTATCGGTAAGTCGTTCATCACATGTGCGTTCGTTGTGTGGTCCTTATGGAGAGACCCTCAGTTGAAGATACTTATCGTATCAGCCTCTAAGG  >  NC_001604.1/37046‑37643`  

                                                                                                                                                                                                                                                                                                            `|`                                                                                                                                                                                                                                                                                                           
      `gAGGCTGCGAAGAGCACTCAAAGAGCAATCGATGCGGTATCTGCTAAGTATCAAGAAGACCTTGCCGCGCTGGAAGGGAGCACTGATAGGATTATTTCTGATTTGCGTAGCGACAATAAGCGGTTGCGCGTCAGAGTCAAAACTACCGGAACCTCCGATGGTCAGTGTGGATTCGAGCCTGATGGTCGAGCCGAACTTGACGACCGAGATGCTAAACGTATTCTCGCAGTGACCCAGAAGGGTGACGCATGGATTCGTGCGTTACAGGATACTATTCGTGAACTGCAACGTAAGCAggctg                                                                                                                                                                                                                                                                                                     >  1:190185/1‑298 (MQ=255)`  
        `ggctcggagatgtgtataagagacagtCGATGCGGTATCTGCTAAGTATCAAGAAGACCTTGCCGCACTGGAAGGGAGCACTGATAGGATTATTTCTGATTTGCGTAGCGACAATAAGCGGTTGCGCGTCAGAGTCAAAACTACCGGAACCTCCGATGGTCAGTGTGGATTCGAGCCTGATGGTCGAGCCGAACTTGACGACCGAGATGCTAAACGTATTCTCGCAGTGACCCAGAAGGGTGACGCATGGATTCGTGCGTTACAGGATACTATTCGTGAACTGCAACGTAAGCAGGAAATc                                                                                                                                                                                                                                                                                                   <  1:213508/275‑1 (MQ=255)`  
                      `cTCAAAGAGCAATCGATGCGGTATCTGCTAAGTATCAAGAAGACCTTGCCGCGCTGGAAGGGAGCACTGATAGGATTATTTCTGATTTGCGTAGCGACAATAAGCGGTTGCGCGTCAGAGTCAAAACTACCGGAACCTCCGATGGTCAGTGTGGATTCGAGCCTGATGGTCGAGCCGAACTTGACGACCGAGATGCTAAACGTATTCTCGCAGTGACCCAGAAGGGTGACGCATGGATTCGTGCGTTACAGGATACTATTCGTGAACTGCAACGTAAGCAGGAAATCAAGTAAGGAGGCaa                                                                                                                                                                                                                                                                                     >  1:7514/1‑301 (MQ=255)`  
                        `cAAAGAGCAATCGATGCGGTATCTGCTAAGTATCAAGAAGACCTTGCCGCGCTGTAAGGGAGCACTTATAGGATTATTTCTGATTTGCGTAGCTACAATAAGCGGTTGCGCGTCAGAGTCAAAACTACCGGAACCTCCGATGGTCAGTGTGGATTCGAGCCTGATGGTCGAGCCGAACTTGACGACCGAGATGCTAAACGTATTCTCGCAGAGACCCAGAAGGGTGACGCATGGATTCGTGCGTTACAGGATACTATTCGTGAACTGCAACGTAAGCAGGAAATCAAGTAAGGAGGCAAtg                                                                                                                                                                                                                                                                                   <  1:54675/301‑1 (MQ=255)`  
                        `aaaaGAGCAATCGAAGCGGGATCTGGTAAGTATCAAGAAGGCCTTGCCGCGCTGGAAGGGAGCACTGATAGGATTATTTCTGAGTTGCGTAGCGACAATAAGCGGTTGCGCGTCAGAGTCAAAACGACCGGAACCTCCGATGGTCAGTGTGGATTCGAGCCTGATGGTCGAGCCGAACTTGACGACCGAGATGCTAAACGTATTCTCGCAGTTACCCAGAAGGGTGACGCATGGATTCGTGCGTTACAGGATACTATTCGTGAACTGCAACGTAAGCAGGAAATCAAGTAAGGAGGCAAtg                                                                                                                                                                                                                                                                                   <  1:255551/300‑1 (MQ=255)`  
                            `gctctgagatgtgtataagagacaggTATCAAGAAGACCTTGCCGCGCTGGAAGGGAGCACTGATAGGATTATTTCTGATTTGCGTAGCGACAATAAGCGGTTGCGCGTCAGAGTCAAAACTACCGGAACCTCCGATGGTCAGTGTGGATTCGAGCCTGATGGTCGAGCCGAACTTGACGACCGAGATGCTAAACGTATTCTCGCAGTGACCCAGAAGGGTGACGCATGGATTCGTGCGTTACAGGATACTATTCGTGAACTGCAACGTAAGCAGGAAATCAAGTAAGGAGGCAATGTGTc                                                                                                                                                                                                                                                                               <  1:49067/276‑1 (MQ=255)`  
                            `gagCAATCGATGCGGTATCTGCTAAGTATCAAGAAGACCTTGCCGCGCTGGAAGGGAGCACTGATAGGATTATTTCTGATTTGCGTAGCGACAATAAGCGGTTGCGCGTCAGAGTCAAAACTACCGGAACCTCCGATGGTCAGTGTGGATTCGAGCCTGATGGTCGAGCCGAACTTGACGACCGAGATGCTAAACGTATTCTCGCAGTGACCCAGAAGGGTGACGCATGGATTCGTGCGGTACAGGATACTATTCGTGAACTGCAACGTAAGCAGGAAATCAAGtctgtctcttatacaca                                                                                                                                                                                                                                                                               >  1:344037/1‑285 (MQ=255)`  
                                 `atggtagCGGTATCTGCAAATGATCAAGAAGAACTTGCCGCGGTGGAAGGGAGCACGGATAGGATTATTTCTGATTTGCGTAGCGACAATAAGCGGTTGCGCGTCAGAGTCAAAACTACCGGAACCTCCGATGGTCAGTGTGGATTCGAGCCTGATGGTCGAGCCGAACTTGACGACCGAGATGCTAAACGTATTCTCGCAGTGACCCAGAAGGGTGACGCATGGATTCGTGCGTTACAGGATACTATTCGTGAACTGCAACGTAAGCAGGAAATCAAGTAAGGAGGCAATGTGTCTACTc                                                                                                                                                                                                                                                                          <  1:220068/295‑1 (MQ=255)`  
                                 `aTCGATGCGGTATCTGCTAAGTATCAAGAAGACCTTGCCGCGCTGGAAGGGAGCACTGATAGGATTATTTCTGATTTGCGTAGCGACAATAAGCGGTTGCGCGTCAGAGTCAAAACTACCGGAACCTCCGATGGTCAGTGTGGATTCGAGCCTGATGGTCGAGCCGAACTTGACGACCGAGATGCTAAACGTATTCTCGCAGTGACCCAGAAGGGTGACGCATGGATTCGTGCGTTACAGGATACTATTCGTGAACTGCAACGTAAGCAGGAAATCAAGTAAGGAGGCAATGTGTCTACTc                                                                                                                                                                                                                                                                          <  1:293399/301‑1 (MQ=255)`  
                                 `aTCGATGCGGTATCTGCTAAGTATCAAGAAGAACTTGCCGCGCTGGAAGGGAGCACTGATAGGATTATTTCTGATTTGCGTAGCGACAATAAGCGGTTGCGCGTCAGAGTCAAAACTACCGGAACCTCCGATGGTCAGTGTGGATTCGAGCCTGATGGTCGAGCCGAACTTGACGACCGAGATGCTAAACGTATTCTCGCAGTGACCCAGAAGGGTGACGCATGGATTCGTGCGTTACAGGATACTATTCGTGAACTGCAACGTAAGCAGGAAATCAAGTAAGGAGGCAATGTGTCTACTc                                                                                                                                                                                                                                                                          <  1:194516/301‑1 (MQ=255)`  
                                      `ggCGGTATCTGCTAAGTATCAAGAAGACCTTGCCGCGCTGGAAGGGAGCACTGATAGGATTATTTCTGATTTGCGTAGCGACAATAAGCGGTTGCGCGTCAGAGTCAAAACTACCGGAACCTGCGATGGTCAGTGTGGATTCGAGCCTGATGGTCGAGCCGAACTTGACGACCGAGATGCTAAACGTATTCTCGCAGTGACCCAGAAGGGTGACGCATGGATTTGTGCGTTACAGGATACTATTCGTGAACTGCAACGTAAGCAGGAAATCAAGTAAGGAGGCAATGTGTCTACTcaatcc                                                                                                                                                                                                                                                                     <  1:100041/300‑1 (MQ=255)`  
                                         `ggTATCTGCTAAGTATTAAGAAGACCTTGCCGAGCTGGAAGGGAGCACTGATAGGATTATTTCTGATTTGCGTAGCGAAAATAAGCGGTTGCGCGTCAGAGTCAAAACTACCGGAACCTCCGATGGTCAGTGTGGATTCGAGCCTGATGGTCGAGCCGAACTTGACGACCGAGATGCTAAACGTATTCTCGCAGTGACCCAGAAGGGTGACGCATGGATTCGTGCGTTACAGGATACTATTCGTGAACTGCAACGTAAGCAGGAAATCAAGTAAGGAGGCAATGTGTCTACTcaatccaat                                                                                                                                                                                                                                                                  <  1:250395/301‑1 (MQ=255)`  
                                           `tgtaaaagagacagacagaagcCCTGGCCGCGCTGGAAGGTAGCACTGATAGGATTATTTCTGATTTGCGTAGCGACAATAAGCGGTTGCGCGTCAGAGTCAAAACTACCGGAACCTCCGATGGTCAGTGTGGATTCGAGCCTGATGGTCGAGCCGAACTTGACGACCGAGATGCTAAACGTATTCTCGCAGTGACCCAGAAGGGTGACGCATGGATTCGTGCGTTACAGGATACTATTCGTGAACTGCAACGTAAGCAGGAAATCAAGTAAGGAGGCAATGTGTCTACTCAATCCAATCg                                                                                                                                                                                                                                                                <  1:295880/285‑1 (MQ=255)`  
                                           `tATCTGCGAAGTATCAAGAAGACCTTGCCGCGATGGAAGGGAGAACTGATAGGATTATTTCTGATTTGCGTAACGACAATAAGCGGTTGCGCGGCAGAGTGAAAACTACCGGAACCTCCGATGGTCAGTGTGGATTCGAGCCTGATGGTCGAGCCGAACTTGACGACCGAGATGCTAAACGTATTCTCGCAGTGACCCAGAAGGGTGACGCATGGATTCGTGCGTTACAGGATACTATTCGTGAACTGCAACGTAAGCAGGAAATCAAGTAAGGAGGCAATGTGTCTACTCAATCCAATCg                                                                                                                                                                                                                                                                <  1:203610/301‑1 (MQ=255)`  
                                              `cggCTAAGTATCAAGAAGGCCTTGCCGGGCTGGAAGGGAGCACTGATAGGATTATTGCTGATTTGCGTAGCGACAATAAGCGGTTGCGCGTCAGAGTCAAAACTACCGGAACCTCCGATGGTCAGTGTGGATTCGAGCCTGATGGTCGAGCCGAACTTGACGACCGAGATGCTAAACGTATTCTCGCAGTGACCCAAAAGGGTGACGCATGGATTCGTGCGTTACAGGATACTATTCGTGAACTGCAACGTAAGCAGGAAATCAAGTAAGGAGGCAATGTGTCTACTCAATCCAATCGTaa                                                                                                                                                                                                                                                             <  1:313643/299‑1 (MQ=255)`  
                                              `cTGCTAAGTATCAAGAAGACCTTGCCGCGCTGGAAGGGAGCACTGATAGGATTATTTCTGATTTGCGTAGCGACAATAAGCGGTTGCGCGTCAGAGTCAAAACTACCGGAACCTCCGATGGTCAGTGTGGATTCGAGCCTGATGGTCGAGCCGAACTTGACGACCGAGATGCTAAACGTATTCTCTCAGTGACCCAGAAGGGTGACGCATGGATTCGTGCGTTACAGGATACTATTCGTGAACTGCAACGTAAGCAGGAAATCAAGTAAGGAGGCAATGTGTCTACTCAATCCAATCGTaa                                                                                                                                                                                                                                                             >  1:319382/1‑301 (MQ=255)`  
                                              `cTGCTAAGTATCAAGAAGACCTTGCCGCGCTGGAAGGGAGCACTGATAGGATTATTTCTGATTTGCGTAGCGACAATAAGCGGGTGCGCGTCAGAGTCAAAACTACCGGAACCTCCGATGGTCAGTGTGGATTCGAGCCTGATGGTCGAGCCGAACTTGACGACCGAGATGCTAAACGTATTCTCGCAGTGACCCAGAAGGGTGACGCATGGATTCGTGCGTTACAGGATACTATTCGTGAACTGCAACGTAAGCAGGAAATCAAGTAAGGAGGCAATGTGTCTACTCAATCCAATCGTaa                                                                                                                                                                                                                                                             >  1:88051/1‑301 (MQ=255)`  
                                                `gCTAAGTATCAAGAAGACCTTGCCTCGCTGGAAGGGAGCACTGATAGGATTATTTCTGATTTGCGTAGCGACAATAAGCGGTTGCGCGTCAGAGTCAAAACTACCGGAACCTCCGATGGTCAGTGTGGATTCGAGCCTGATGGTCGAGCCGAACTTGACGACCGAGATGCTAAACGTATTCTCGCAGTGACCCCGACGGGTGACGCATGGATTCGTGCGTTACAGGATACTATTCGTGCACTGCAACGTAAGCAGGAAATCAAGTAAGGAGGCAATGTGTCTACTCAATCCAATCGTAAtt                                                                                                                                                                                                                                                           >  1:29024/1‑300 (MQ=255)`  
                                                     `gTATCAAGAAGACCTTGCCGCGCTGGAAGGGAGCACTGATAGGATTATTTCTGATTTGCGTAGCGACAATAAGCGGTTGCGCGTCAGAGTCAAAACTACCGGAACCTCCGATGGTCAGTGTGGATTCGAGCCTGATGGTCGAGCCGAACTTGACGACCGAGATGCTAAACGTATTCTCGCAGTGACCCAGAAGGGTGACGCATGGATTCGTGCGTTACAGGATACTATTCGTGAACTGCAACGTAAGCAGGAAATCAAGTAAGGAGGCAATGTGTCTACTCAATCCAATCGTAATGCGCTc                                                                                                                                                                                                                                                      >  1:304894/1‑301 (MQ=255)`  
                                                         `cAAGAAGACCTTGCCGCGCTGGAAGGGAGCACTGATAGGATTATTTCTGATTTGCGTAGCGACAATAAGCGGTTGCGCGTCAGAGTCAAAACTACCGGAACCTCCGATGGTCAGTGTGGATTCGAGCCTGATGGTCGAGCCGAACTTGACGACCGAGATGCTAAACGTATTCTCGCAGTGACCCAGAAGGGTGACGCATGGATTCGTGCGTTACAGGATACTATTCGTGAACTGCAACGTAAGCAGGAAATCAAGTAAGGAGGCAATGTGTCTACTCAATCCAATCGTAATGCGCTCGTAg                                                                                                                                                                                                                                                  >  1:209593/1‑301 (MQ=255)`  
                                                           `aagataCCTTGCCGCGCTGGAAGGGAGCACTGATAGGATTATTTCTGATTTGCGTAGCGACAATAAGCGGTTGAGCGTCAGAGTCAAAACTACCGGAACCTCCGATGGTCAGTGTGGATTCGAGCCTGATGGTCGAACCGAACTTGACGACCGAGATGCTAAACGTATTCTCGCAGTGACCCAGAAGTGTGACGCATGGATTCGTGCGTTACAGGATACTATTCGTGAACTGCAACGTAAGCAGGAAATCAAGTAAGGAGGCAATGTGTCTACTCAATCCAATCGTAATGCGCTCGTAGTg                                                                                                                                                                                                                                                <  1:102696/296‑1 (MQ=255)`  
                                                                       `cgcgCTGGAAGGGAGCACTGATAGGATTATTTCTGATTTGCGTAGCGACAATAAGCGGTTGCGCGTCAGAGTCAAAACTACCGGAACCTCCGATGGTCAGTGTGGATTCGAGCCTGATGGTCGAGCCGAACTTGACGACCGAGATGCTAAACGTATTCTCGCAGTGACCCAGAAGGGTGACGCATGGATTCGTGCGTTACAGGATACTATTCGTTAACTGCAACGTAAGCAGGAAATCAAGTAAGGAGGCAATGTGTCTACTCAATCCAATCGTAATGCGCTCGTAGTGGCGCAACTGaaa                                                                                                                                                                                                                                    <  1:155658/301‑1 (MQ=255)`  
                                                                           `cTGGAAGGGAGCACTGATAGGATTATTTCTGATTTGCGTAGCGACAATAAGCGGTTGCGCGTCAGAGTCAAAACTACCGGAACCTCCGATGGTCAGTGTGGATTCGAGCCTGATGGTCGAGCCGAACTTGACGACCGAGATGCTAAACGTATTCTCGCAGTGACCCAGGAGGGTGACGCATGGATTCGTGCGTTACAGGATACTATTCGTGAACTGCAACGTAAGCAGGAAATCAAGTAAGGAGGCAATGTGTCTACTCAATCCAATCGTAATGCGCTCGTAGTGGCGCAACTGAAAGgag                                                                                                                                                                                                                                >  1:223516/1‑301 (MQ=255)`  
                                                                             `gtataagagacaggATAGGATGATTTCTGATTGGCGTAGCGACAATAAGCGGTGGCGCGTCAGAGTCAAAACTACCGGAACCTCCGATGGTCAGTGTGGATTCGAGCCTGATGGTCGAGCCGAACTTGACGACCGAGATGCTAAACGTATTCTCGCAGTGACCCAGAAGGGTGACGCATGGATTCGTGCGTTACAGGATACTATTCGTGAACTGCAACGTAAGCAGGAAATCAAGTAAGGAGGCAATGTGTCTACTCAATCCAATCGTAATGCGCTCGTAGTGGCGCAACTGAAAGGAGAc                                                                                                                                                                                                                              <  1:310562/288‑1 (MQ=255)`  
                                                                             `ggAAGTGAGCACTGATAGGATTATTTCTGATTTGCGTAGCGACAATAAGCGGTTGCGCGTCAGAGTCAAAACTACCGGAACCTCCGATGGTCAGTGTGGATTCGAGCCTGATGGTCGAGCCGAACTTGACGACCGAGATGCTAAACGTATTCTCGCAGTGACCCAGAAGGGTGACGCATGGATTCGTGCGTTACAGGATACTATTCGTGAACTGCAACGTAAGCAGGAAATCAAGTAAGGAGGCAATGTGTCTACTCAATCCAATCGTAATGCGCTCGTAGTGGCGCAACTGAAAGGAGAc                                                                                                                                                                                                                              <  1:179132/301‑1 (MQ=255)`  
                                                                             `ggAAGGGAGCACTGATAGGATTATTTCTGATTTGCGTAGCGACAATAAGCGGTTGCGCGTCAGAGTCAAAACTACCGGAACCTCCGATGGTCAGTGTGGATTCGAGCCTGATGGTCGAGCCGAACTTGACGACCGAGATGCTAAACGTATTCTCGCAGTGACCCAGAAGGGTGACGCATGGATTCGTGCGTTACAGGATACTATTCGTGAACTGCAACGTAAGCAGGAAATCAAGTAAGGAGGCAATGTGTCTACTCAATCCAATCGTAATGCGCTCGTAGTGGCGCAACTGAAAGGAGAc                                                                                                                                                                                                                              <  1:111475/301‑1 (MQ=255)`  
                                                                             `ggAAGGGAGCACTGATAGGATTATTTCTGATTTGCGTAGCGACAATAAGCGGTTGCGCGTCAGAGTCAAAACTACCGGAACCTCCGATGGTCAGTGTGGATTCGAGCCTGATGGTCGAGCCGAACTTGACGACCGAGATGCTAAACGTATTCTCGCAGTGACCCAGAAGGGTGACGCATGGATTCGTGCGTTACAGGATACTATTCGTGAACTGCAACGTAAGCAGGAAATCAAGTAAGGAGGCAATGTGTCTACTCAATCCAATCGTAATGCGCTCGTAGTGGCGCAACTGAAAGGAGAc                                                                                                                                                                                                                              <  1:120199/301‑1 (MQ=255)`  
                                                                             `ggAAGGGAGCACTGATAGGATTATTTCTGATTTGCGTAGCGACAATAAGCGGTTGCGCGTCAGAGTCAAAACTACCGGAACCTCCGATGGTCAGTGTGGATTCGAGCCTGATGGTCGAGCCGAACTTGACGACCGAGATGCTAAACGTATTCTCGCAGTGACCCAGAAGGGTGACGCATGGATTCGTGCGTTACAGGATACTATTCGTGAACTGCAACGTAAGCAGGAAATCAAGTAAGGAGGCAATGTGTCTACTCAATCCAATCGTAATGCGCTCGTAGTGGCGCAACTGAAAGGAGAc                                                                                                                                                                                                                              >  1:158345/1‑301 (MQ=255)`  
                                                                             `ggAAGGGAGCACTGATAGGATTATTTCTGATTTGCGTAGCGACAATAAGCGGTTGCGCGTCAGAGTCAAAACTACCGGAACCTCCGATGGTCAGTGTGGATTCGAGCCTGATGGTCGAGCCGAACTTGACGACCGAGATGCTAAACGTATTCTCGCAGTGACCCAGAAGGGTGACGCATGGATTCGTGCGTTACAGGATACTATTCGCGAACTGCAACGTAAGCAGGAAATCAAGTAAGGAGGCAATGTGTCTACTCAATCCAATCGTAATGCGCTCGTAGTGGCGCAACTGAAAGGAGAc                                                                                                                                                                                                                              >  1:302371/1‑301 (MQ=255)`  
                                                                             `ataagagacagcTGATAGGATTATTGCTGATTTGCGTAGCGACAATAAGCGGTTGCGCGTCAGAGTCAAAACTACCGGAACCTCCGATGGTCAGTGTGGATTCGAGCCTGATGGTCGAGCCGAACTTGACGACCGAGATGCTAAACGTATTCTCGCAGTGACCCAGAAGGGTGACGCATGGATTCGTGCGTTACAGGATACTATTCGTGAACTGCAACGTAAGCAGGAAATTAAGTAAGGAGGCAATGTGTCTACTCAATCCAATCGTAATGCGCTCGTAGTGGCGCAACTGAAAGGAGAc                                                                                                                                                                                                                              <  1:206379/290‑1 (MQ=255)`  
                                                                              `gAAGGGAGCACTGATAGGATTATTTCTGATTTGCGTAGCGACAATAAGCGGTTGCGCGTCAGAGTCAAAACTACCGGAACCTCCGATGGTCAGTGTGGATTCGAGCCTGATGGTCGAGCCGAACTTGACGACCGAGATGCTAAACGTATTCTCGCAGTGACCCAGAAGGGTGACGCATGGATTCGTGCGTTACAGGATACTATTCGTGAACTGCAACGTAAGCAGGAAATCAAGTAAGGAGGCAATGTGTCTACTCAATCCAATCGTAATGCGCTCGTAGTGGCGCAACTGAAAggctgtc                                                                                                                                                                                                                             >  1:326479/1‑296 (MQ=255)`  
                                                                                `aGGGAGCACTGATAGGATTATTTCTGATTTGCGTAGCGACAATAAGCGGTTGCTCGTCAGAGTCAAAACTACCGGAACCTCCGATGGTCAGTGTGGATTCGAGCCTGATGGTCGAGCCGAACTTGACGACCGAGATGCTAAACGTATTCTCGCAGTGACCCAGAAGGGTGACGCATGGATTCGTGCGTTACAGGATACTATTCGTGAACTGCACCGTAAGTAGGAAATCAAGTAAGGAGGCAATGTGTATACTCAATCCACTCGTAATGCGCTCGTATTGGCGCAACTGAAAGGTGACttt                                                                                                                                                                                                                           >  1:974/1‑300 (MQ=255)`  
                                                                                `aGGGAGCACTGATAGGATTATTTCTGATTTGCGTAGCGACAATAAGCGGTTGCGCGTCAGAGTCAAAACTACCGGAACCTCCGATGGTCAGTGTGGATTCGAGCCTGATGGTCGAGCCGAACTTGACGACCGAGATGCTAAACGTATTCTCGCAGTGACCCAGAAGGGTGACGCATGGATTCGTGCGTTACAGGATACTATTCGTGAACTGCAACGTAAGCAGGAAATCAAGTAAGGAGGCAATGTGTCTACTCAATCCAATCGTAATGCGCTCGTAGTGGCGCAACTGAAAggctgtcgc                                                                                                                                                                                                                           >  1:268830/1‑294 (MQ=255)`  
                                                                                      `cACTGATAGGATTATTTCTGATTTGCGTAGCGACAATAAGCGGTTGCGCGTCAGAGTCAAAACTACCGGAACCTCCGATGGTCAGTGTGGATTCGAGCCTGATGGTCGAGCCGAACTTGACGACCGAGATGCTAAACGTATTCTCGCAGTGACCCAGAAGGGTGACGCATGGATTCGTGCGTTACAGGATACTATTCGTGAACTGCAACGTAAGCAGGAAATCAAGTAAGGAGGCAATGTGTCTACTCAATCCAATCGTAATGCGCTCGTAGTGGCGCAACTGAAAGGAGACTTCGTGGCg                                                                                                                                                                                                                     <  1:7249/301‑1 (MQ=255)`  
                                                                                            `tAGGATTATTTCTGATTTGCGTAGCGACAATAAGCGGTTGCGCGTCAGAGTCAAAACTACCGGAACCTCCGATGGTCAGTGTGGATTCGAGCCTGATGGTCGAGCCGAACTTGACGACCGAGATGCTAAACGTATTCTCGCAGTGACCCAGAAGGGTGACGCATGGATTCGTGCGTTACAGGATACTATTCGTGAACTGCAACGTAAGCAGGAAATCAAGTAAGGAGGCAATGTGTCTACTCAATCCAATCGTAATGCGCTCGTAGTGGCGCAACTGAAAGGAGAATTCGTGGCGTTACtt                                                                                                                                                                                                               >  1:170934/1‑300 (MQ=255)`  
                                                                                                        `gtgtataagggacagtgagaggaggTTGCGCGGCAGAGTAAAAACTTACGGAACCTCCTATGGTCAGTGTGGATTCGAGCTGGATTGTCGAGCCGAACGTGACGATCGAGATGCTACACGTATTCTCGCAGTGACCCAGAAGGGTGACGCATGTATTCGTGCGGTACAGGATACTATTCGTGAACTGAAACGTAAGCAGGAAATCAAGTAAGGAGGCAATGTGTCTACTCAATTCAATCGTAATGCGCTCGTAGTGGCGCAACTGAAAGGAGAATTCGTGGCGTTCCTATTCGTCTTATgg                                                                                                                                                                                                   <  1:30026/278‑1 (MQ=255)`  
                                                                                                              `gCGTAGCGACAATAAGCGGTTGCGCGTCAGAGTCAAAACTACCGGAACCTCCGATGGTCAGTGTGGATTCGAGCCTGATGGTCGAGCCGAACTTGACGACCGAGATGCTAAACGTATTCTCGCAGTGACCCAGAAGGGTGACGCATGGATTCGTGCGTTACAGGATACTATTCGTGAACTGCAACGTAAGCAGGAAATCAAGTAAGGAGGCAATGTGTCTACTCAATCCAATCGTAATGCGCTCGTAGTGGCGCAACTGAAAGGAGACTTCGTGGCGTTccctgtctcttatacacatctc                                                                                                                                                                                             >  1:17682/1‑281 (MQ=255)`  
                                                                                                              `gCGTAGCGACAATAAGCGGTTGCGCGTCAGAGTCAAAACTACCGGAACCTCCGATGGTCAGTGTGGATTCGAGCCTGATGGTCGAGCCGAACTTGACGACCGAGATGCTAAACGTATTCTCGCAGTGACCCAGAAGGGTGACGCATGGATTCGTGCGTTACAGGATACTATTCGTGAACTGCAACGTAAGCAGGAAATCAAGTAAGGAGGCAATGTGTCTACTCAATCCAATCGTAATGCGCTCGTAGTGGCGCAACTGAAAGGAGACTTCGTGGCGTTCCTATTCGTCTTATGGAAGgct                                                                                                                                                                                             >  1:307857/1‑300 (MQ=255)`  
                                                                                                                    `ggACAATAAGCGGTTGCGCGTCAGAGTCAAAACTACCGGAACCTCCGATGGTCAGTGTGGATTCGAGCCTGATGGTCGAGCCGAACTTGACGACCGAGATGCTAAACGTATTCTCGCAGTGACCCAGAAGGGTGACGCATGGATTCGTGCGTTACAGGATACTATTCGTGAACTGCAACGTAAGCAGGAAATCAAGTAAGGAGGCAATGTGTCTACTCAATCCAATCGTAATGCGCTCGTAGTGGCGCAACTGAAAGGAGACTTCGTGGCGTTCCTATTCGTCTTATGGAAGGCGCTAAAc                                                                                                                                                                                       <  1:282397/300‑1 (MQ=255)`  
                                                                                                                       `cAATAAGCGGTTGCGCGTCAGAGTCAAAACTACCGGAACCTCCGATGGTCAGTGTGGATTCGAGCCTGATGGTCGAGCCGAACTTGACGCCCGAGATGCTAAACGTATTCTCGCAGTGACCCAGAAGGGTGACGCATGGATTCGTGCGTTACAGGCTACTATTCGTGAACTGCAACGTAAGCAGTAAATCAAGTAAGGAGGCAATGTGTCTACTCAATCCAATCGTAATGCGCTCGTAGTGGCGCAACTGAAAGGAGACTTCGTGGCGTTCCTATTCGTCTTATGGAAGGCGCTCAACCTa                                                                                                                                                                                    >  1:340778/1‑301 (MQ=255)`  
                                                                                                                        `aaTAAGCGGTTGCGCGTCAGAGTCAAAACTACCGGAACCTCCGATGGTCAGTGTGGATTCGAGCCTGATGGTCGAGCCGAACTTGACGACCGAGATGCTAAACGTATTCTCGCAGTGACCCAGAAGGGTGACGCATGGATTCGTGCGTTACAGGATACTATTCGTGAACTGCAACGTAAGCAGGAAATCAAGTAAGGAGGCAATGTGTCTACTCAATCCAATCGTAATGCGCTCGTAGTGGCGCAACTGAAAGGAGACTTCGTGGCGTTCCTATTCGTcctgtctcttatacacatctccg                                                                                                                                                                                   >  1:278325/1‑279 (MQ=255)`  
                                                                                                                              `cGGTTGCGCGTCAGAGTCAAAACTACCGGAACCTCCGATGGTCAGTGTGGATTCGAGCCTGATGGTCGAGCCGAACTTGACGACCGAGATGCTAAACGTATTCTCGCAGTGACCCAGAAGGGTGACGCATGGATTCGTGCGTTACAGGATACTATTCGTGAACTGCAACGTAAGCAGGAAATCAAGTAAGGAGGCAATGTGTCTACTCAATCCAATCGTAATGCGCTCGTAGTGGCGCAACTGAAAGGAGACTTCGTGGCGTTCCTATTCGTCTTATGGAAGGCGCTAAAccctgtctctt                                                                                                                                                                             >  1:261170/1‑292 (MQ=255)`  
                                                                                                                              `cGGTTGCGCGTCAGAGTCAAAACTACCGGAACCTCCGATGGTCAGTGTGGATTCGAGCCTGATGGTCGAGCCGAACTTGACGACCGAGATGCTAAACGTATTCTCGCAGTGACCCAGAAGGGTGACGCATGGATTCGTGCGTTACAGGATACTATTCGTGAACTGCAACGTAAGCAGGAAATCAAGTAAGGAGGCAATGTGTCTACTCAATCCAATAGTAATGCGCTCGTAGTGGCGCAACTGAAAGGAGACTTCGTGGCGTTCCTATTCGTCTTATGGAAGGCGCTAAACCTACCggctg                                                                                                                                                                             >  1:70051/1‑298 (MQ=255)`  
                                                                                                                                          `agagTCAAAACTACCGGAACCTCCGATGGTCAGTGTGGATTCGAGCCTGATGGTCGAGCCGAACTTGACGACCGAGATGCTAAACGTATTCTCGCAGTGACCCAGAAGGGTGACGCATGGATTCGTGCGTTACAGGATACTATTCGTGAACTGCAACGTAAGCAGGAAATCAAGTAAGGAGGCAATGTGTCTACTCAATCCAATCGTAATGCGCTCGTAGTGGCGCAACTGAAAGGAGACTTCGTGGCGTTCCTATTCGTCTTATGGAAGGCGCTAAACCTACCGGTGCCCACTAAGTGTc                                                                                                                                                                 <  1:264682/301‑1 (MQ=255)`  
                                                                                                                                             `gTCAAAACTACCGGAACCTCCGATGGTCAGTGTGGATTCGAGCCTGATGGTCGAGCCGAACTTGACGACCGAGATGCTAAACGTATTCTCGCAGTGACCCAGAAGGGTGACGCATGGATTCGTGCGTTACAGGATACTATTCGTGAACTGCAACGTAAGCAGGAAATCAAGTAAGGAGGCAATGTGTCTACTCAATCCAATCGTAATGCGCTCGTAGTGGCGCAACTGAAAGGAGACTTCGTGGCGTTCCTATTCGTCTTATGGAAGGCGCTAAACCTACCGGTGCCCACTAAGTGTCAGa                                                                                                                                                              >  1:196574/1‑301 (MQ=255)`  
                                                                                                                                                  `aaCTACCGGAACCTCCGAGGGGCAGTGTGGATGCGAGCCTGATGGTCGATCCGAACTTGACGACCGAGATGCTAAACGGATTCTCGCAGTGACCCAGAAGGGTGACGCATGGATGCGTGCGTTACAGGTTACTATTCGTGAACTGCAACGTAAGCAGGAAATCAAGTAAGGAGGCAATGTGTCTACTCAATCCAATCGTAATGCGCTCGTAGTGGCGCAACTGAAAGGAGACTTCGTGGCGTTCCTATTCGTCTTATGGAAGGCGCTAAACCTACCGGTGCCCACTAAGTGTCAGATTGAc                                                                                                                                                         <  1:108092/301‑1 (MQ=255)`  
                                                                                                                                                    `cTACCGGAACCTCCGATGGTCAGTGTGGATTCGAGCCTGATGGTCGAGCCGAACTTGACGACCGAGATGCTAAACGTATTCTCGCAGTGACCCAGAAGGGTGACGCATGGATTCGTGCGTTACAGGATACTATTCGTGAACTGCAACGTAAGCAGGAAATCAAGTAAGGAGGCAATGTGTCTACTCAATCCAATCGTAATGCGCTCGTAGTGGCGCAACTGAAAGGAGACTTCGTGGCGTTCCTATTCGTCTTATGGAAGGCACTAAACCTACCGGTGCCCACTAAGTGTCAGATTGACAt                                                                                                                                                       >  1:24739/1‑301 (MQ=255)`  
                                                                                                                                                      `aCCGGAACCTCCGATGGTCAGTGTGGATTCGAGCCTGATGGTCGAGCCGAACTTGACGACCGAGATGCTAAACGTATTCTCGCAGTGACCCAGAAGGGTGACGCATGGATTCGTGCGTTACAGGATACTATTCGTGGACTGCAACGTAAGCAGGAAATCAAGTAAGGAGGCAATGTGTCTACTCAATCCAATCGTAATGCGCTCGTAGTGGCGCAACTGAAAGGAGACTTCGTGGCGTTCCTATTCGTCTTATGGAAGGCGCTAAACCTACCGGTGCCCACTAAGTGTCCGAtctgtctct                                                                                                                                                     >  1:91331/1‑293 (MQ=255)`  
                                                                                                                                                        `cGGAACCTCCGATGGGCAGGGTGGATTCGAGCCTGATGGTCGAGCCGAACTTGACGACCGAGATGCTAAACGTATTCTCGCAGTGACCCAGAAGGGTGACGCATGGATTCGTGCGTTACAGGATACTATTCGTGAACTGCAACGTAAGCAGGAAATCAAGTAAGGAGGCAATGTGTCTACTCAATCCAATCGTAATGCGCTCGTAGTGGCGCAACTGAAAGGAGACTTCGTGGCGTTCCTATTCGTCTTATGGAAGGCGCTAAACCTACCGGTGCCCACTAAGTGTCAGATTGACATGGCt                                                                                                                                                   <  1:45913/301‑1 (MQ=255)`  
                                                                                                                                                             `ccTCCGATGGTCAGTGTGGATTCGAGCCTGATGGTCGAGCCGAACTTGACGACCGAGATGCTAAACGTATTCTCGCAGTGACCCAGAAGGGTGACGCATGGATTCGTGCGTTACAGGATACTATTCGTGAACTGCAACGTAAGCAGGAAATCAAGTAAGGAGGCAATGTGTATACTCAATCCAATCGTAATGCGCTCGTAGTGGCGCAACTGAAAGGAGACTTCGTGGCGTTCCTATTCGTCTTATGGAAGTCGCTAAACCTACCGGTGCCCACTAAGTGTCAGATTGACATGGCTAAGGt                                                                                                                                              <  1:187292/301‑1 (MQ=255)`  
                                                                                                                                                                          `gtgtGGATTCGAGCCTGATGGTCGAGCCGAACTTGACGACCGAGATGCTAAACGTATTCTCGCAGTGACCCAGAAGGGTGACGCATGGATTCGTGCGTTACAGGATACTATTCGTGAACTGCAACGTAAGCAGGAAATCAAGTAAGGAGGCAATGTGTCTACTCAATCCAATCGTAATGCGCTCGTAGTGGCGCAACTGAAAGGAGACTTCGTGGCGTTCCTATTCGTCTTATGGAAGGCGCTAAACCTACCGGTGCCCACTAAGTGTCAGATTGACATGGCTAAGGTGCTGGCGAATGga                                                                                                                                 >  1:161600/1‑301 (MQ=255)`  
                                                                                                                                                                          `gtgtGGATTCGAGCCTGATGGTCGAGCCGAACTTGACGACCGAGATGCTAAACGTATTCTCGCAGTGACCCAGAAGGGTGACGCATGGATTCGTGCGTTACAGGATACTATTCGTGAACTGCAACGTAAGCAGGAAATCAAGTAAGGAGGCAATGTGTCTACTCAATCCAATCGTAATGCGCTCGTAGTGGCGCAACTGAAAGGAGACTTCGTGGCGTTCCTATTCGTCTTATGGAAGGCGCTAAACCTACCGGTGCCCACTAAGTGTCAGATTGACATGGCTAAGGTGCTGGCGAATGga                                                                                                                                 >  1:173827/1‑301 (MQ=255)`  
                                                                                                                                                                               `gATTCGAGCCTGATGGTCGAGCCGAACTTGACGACCGAGATGCTAAACGTATTCTCGCAGTGACCCAGAAGGGTGACGCATGGATTCGTGCGTTACAGGATACTATTCGTGAACTGCAACGTAAGCAGGAAATCAAGTAAGGAGGCAATGTGTCTACTCAATCCAATCGTAATGCGCTCGTAGTGGCGCAACTGAAAGGAGACTTCGTGGCGTTCCTATTCGTCTTATGGAAGGCGCTAAACCTACCGGTGCACACTAAGTGTAAGATTGACATGGCTAAGGTGCTGGCGAATGGAGagaa                                                                                                                            >  1:64808/1‑301 (MQ=255)`  
                                                                                                                                                                                     `aGCCTGATGGTCGAGCCGAACTTGACGACCGAGATGCTAAACGTATTCTCGCAGTGACCCAGAAGGGTGACGCATGGATTCATGCGTTACAGGATACTATTCGTGAACTGCAACGTAAGCAGGAAATCAAGTAAGGAGGCAATGTGTCTACTCAATCCAATCGTAATGCGCTCGTAGTGGCGCAACTGAAAGGAGACTTCGTGGCGTTCCTATTCGTCTTATGGAAGGCACTAAAACTACCGGTGCCCACTAAGTGTCAGATTGACATGGCTAAGGTGCTGGCGAATGGAGATAACaagaa                                                                                                                      >  1:58179/1‑301 (MQ=255)`  
                                                                                                                                                                                      `tgggagatgtgtatgagagacagacgatCGAGATGCTAAACGTATTCACGAAGTGACCCAGAAGGGTAACGCATGGATTCGTGCGTTACAGGATACTATTCGTGAACTGCAACGTAAGCAGGAAATCAAGTAAGGAGGCAATGTGTCTACTCAATCCAAGCGTAATGCGCTCGTAGTGGCGCAACTGAAAGGAGACTTCGTGGCGTTCCTATTCGTCTTATGGAAGGCGCTAAACCTACCGGTGCCCACTAAGTGTCAGATTGACATGGCTAAGGTGCTGGCGAATGGAGACAACaagaag                                                                                                                     <  1:253069/279‑1 (MQ=255)`  
                                                                                                                                                                                            `gggTCGAGCCGAACTTGACGACCGAGATGCTAAACGTATTCTCGCAGTGACCCAGAAGGGTGACGCATGGATTCGTGCGTTACAGGATACTATTCGTGAACTGCAACGTAAGCAGGAAATCAAGTAAGGAGGCAATGTGTCTACTCAATCCAATCGTAATGCGCTCGTAGTGGCGCTACTGAAAGGAGACTTCGTGGCGTTCCTATTCGTCTTATGGAAGGCGCTAAACCTACCGGTGCCCACTAAGTGTCAGATTGACATGGCTAAGGTGCTGGCGAATGGAGACAACAAGAAGTTCATc                                                                                                               <  1:95769/300‑1 (MQ=255)`  
                                                                                                                                                                                              `gttgAGCCGAACTTGACGACCGAGATGCTAAACGTATTCTCGCAGTGACCCAGAAGGGTGACGCATGGATTCGTGCGTTACAGGATACTATTCGTGAACTGCAACGTAAGCAGGAAATCAAGTAAGGAGGCAATGTGTCTACTCAATCCAATCGTAATGCGCTCGTAGTGGCGCAACTGAAAGGAGACTTCGTGGCGTTCCTATTCGTCTTATGGAAGGCGCTAAACCTACCGGTGCCCACTAAGTGTCAGATTGACATGGCTAAGGTGCTGGCGAATggctgtctcttatacacatctcc                                                                                                             >  1:271365/4‑280 (MQ=255)`  
                                                                                                                                                                                                  `aGCCGAACTTGACGACCGAGATGCTAAACGTATTCTCGCAGTGACCCAGAAGTGTGACGCATTGATTCGTGCGTTACAGGATACTATTCGTGAACTGCAACGTAAGCAGGAAATCAAGTAAGGAGGCAATGTGTCTACTCAATCCAATCGTAATGCGCTCGTAGTGGCGCAACTGAAAGGAGACTTCGTGGCGTTCCTATTCGTCTTATGGAAGGCGCTAAACCTACCGGTGCCCACTAAGTGTCAGATTGACATGGCTAAGGTGCTGGCGAATGGAGACAACAAGAAGTTCATCTTACAg                                                                                                         >  1:85423/1‑301 (MQ=255)`  
                                                                                                                                                                                                  `aGCCGAACTTGACGACCGAGATGCTAAACGTATTCTCGCAGTGACCCAGAAGGGTGACGCATGGATTCGTGCGTTACAGGATACTATTCGTGAACTGCAACGTAAGCAGGAAATCAAGTAAGGAGGCAATGTGTCTACTCAATCCAATCGTAATGCGCTCGTAGTGGCGCAACTGAAAGGAGACTTCGTGGCGTTCCTATTCGTCTTATGGAAGGCGCTAAACCTACCGGTGCCCACTAAGTGTCAGATTGACATGGCTAAGGTGCTGGCGAATGGAGACAACAAGAAGTTCATCTTACAg                                                                                                         >  1:105068/1‑301 (MQ=255)`  
                                                                                                                                                                                                          `ttGACGACCGAGATGCTAAACGTATGCTCGCAGTGACCCAGAAGGGTGACGCATGGAGTCGTGCGTTACAGGATACTATTCGTGAACTGCAACGTAAGCAGGAAATCAAGTAAGGAGGCAATGTGTCTACTCAATCCAATCGTAATGCGCTCGTAGTGGCGCAACTGAAAGGAGACTTCGTGGCGTTCCTATTCGTCTTATGGAAGGCGCTAAACCTACCGGTGCCCACTAAGTGTCAGATTGACATGGCTAAGGTGCTGGCGAATGGAGACAACAAGAAGTTCATCTTACAGGCTTTCCg                                                                                                 <  1:129257/301‑1 (MQ=255)`  
                                                                                                                                                                                                                 `ccGAGATGCTAAACGTATTCTCGCAGTGACCCAGAAGGGTGACGCATGGATTCGTGCGTTACAGGATACTATTCGTGAACTGCAACGTAAGCAGGAAATCAAGTAAGGAGGCAATGTGTCTACTCAATCCAATCGTAATGCGCTCGTAGTGGCGCAACTGAAAGGAGACTTCGTGGCGTTCCTATTCGTCTTATGGAAGGCGCTAAACCTACCGGTTCCCACTAAGTGTCAGATTGACATGGCTAAGGTGCTGGCGAATGGAAACACCAAGAGGGTCAGCTTACAGGCTTTcattgggatg                                                                                          >  1:184062/1‑292 (MQ=255)`  
                                                                                                                                                                                                                      `aTGCTAAACGTATTCTCGCAGTGACCCAGAAGGGTGACGCATGGATTCGTGCGTTACAGGATACTATTCGTGAACTGCAACGTAAGCAGGAAATCAAGTAAGGAGGCAATGTGTCTACTCAATCCAATCGTAATGCGCTCGTAGTGGCGCAACTGAAAGGAGACTTCGTGGCGTTCCTATTCGTCTTATGGAAGGCGCTAAACCTACCGGTGCCCACTAAGTGTCAGATTGACATGGCTAAGGTGCTGGCGAATGGAGACAACAAGAAGTTCATCTTACAGGCttctgtctcttatacaca                                                                                     >  1:148206/1‑285 (MQ=255)`  
                                                                                                                                                                                                                           `aaaCGTATTCTCGCAGTGACCCAGAAGGGTGACGCATGGATTCGTGCGTTACAGGATACTATTCGTGAACTGCAACGTAAGCAGGAAATCAAGTAAGGAGGCAATGTGTCTACTCAATCCAATCGTAATGCGCTCGTGGTGGCGCAACTGAAAGGAGACTTCGTGGCGTTCCTATTCGTCTTCTGGAAGGCGCTAAACCTACCTGTGCCCACTAAGTGTCATATTGACATGGCTAAGGTGCTGGCGAATGGAGACAACAAGAAGTTCATCTTACAGGCTTTCCGTGGTATCGGTAAGTCGt                                                                                >  1:142477/1‑301 (MQ=255)`  
                                                                                                                                                                                                                            `aaCGTATTCTCGCAGTGACCCAGAAGGGTGACGCATGGATTCGTGCGTTACAGGATACTATTCGTGAACTGCAACGTAAGCAGGAAATCAAGTAAGGAGGCAATGTGTCTACTCAATCCAATCGTAATGCGCTCGTAGTGGCGCAACTGAAAGGAGACTTCGTGGCGTTCCTATTCGTCTTATGGAAGGCGCTAAACCTACCGGTGCCCACTAAGTGTCAGATTGACATGGCGAAGGTGCTGGCGAATGGAGACAACAAGAAGGTCATCTTACAGGCTTTCCGTGGTATCGGTAAGTCGtt                                                                               >  1:216957/1‑301 (MQ=255)`  
                                                                                                                                                                                                                              `cGTATTCTCGCAGTGACCCAGAAGGGTGACGCATGGATTCGTGCGTTACAGGATACTATTCGTGAACTGCAACGTAAGCAGGAAATCAAGTAAGGAGGCAATGTGTCTACTCAATCCAATCGTAATGCGCTCGTAGTGGCGCAACTGAAAGGAGACTTCGTGGCGTTCCTATTCGTCTTATGGAAGGCGCTAAACCTACCGGTGCCCACTAAGTGTCAGATTGACATGGCTAAGGTGCTGGCGAATGGAGACAACAAGAAGTTCATCTTACAGGCTTTCCGTGGTATCGGTAAGTCGTtca                                                                             >  1:329263/1‑301 (MQ=255)`  
                                                                                                                                                                                                                              `cGTATTCTCGCAGTGACCCAGAAGGGTGACGCATGGATTCGTGCGTTACAGGATACTATTCGTGAACTGCAACGTAAGCAGGAAATCAAGTAAGGAGGCAATGTGTCTACTCAATCCAATCGTAATGCGCTCGTAGTAGCGCAACTGAAAGGAGACTTCGTGGCGTTCCTATTCGTCTTATGGAAGGCGCTAAACCTACCGGTGCCCACTAAGTGTCAGATTGACATGGCTAAGGTGCTGGCGAATGTAGACAACAAGAAGTTCATCTTACAGGCTTTCCGtctgtctcttatacacatct                                                                             >  1:145209/1‑282 (MQ=255)`  
                                                                                                                                                                                                                                 `tataagagacagACCCAGAAGGGTGACGCATGGATTCGTGCGTTACAGGATACTATTCGTGAACTGCAACGTAAGCAGGAAATCAAGTAAGGAGGCAATGTGTCTACTCAATCCAATCGTAATGCGCTCGTAGTGGCGCAACTGAAAGGAGACTTCGTGGCGTTCCTATTCGTCTTATGGAAGGCGCTAAACCTACCGGTGCCCACTAAGTGTCAGATTGACATGGCTAAGGTGCTGGCGAATGGAGACAACAAGAAGTTCATCTTACAGGCTTTCCGTGGTATCGGTAAGTCGTtcatca                                                                          <  1:127090/290‑1 (MQ=255)`  
                                                                                                                                                                                                                                  `ttCTCGCAGTGACCCAGAAGGGTGACGCATGGATTCGTGCGTTACAGGATACTATTCGTGAACTGCAACGTAAGCAGGAAATCAAGTAAGGAGGCAATGTGTCTACTCAATCCAATCGTAATGCGCTCGTAGTGGCGCAACTGAAAGGAGACTTCGTGGCGTTCCTATTCGTCTTATGGAAGGCGCTAAACCTACCGGTGCCCACTAAGTGTCAGATTGACATGGCTAAGGTGCTGGCGAATGGAGACAACAAGAAGTTCATCTTACAGGCTTTCCGTGGTATCGGTAAGTCGTTCATcac                                                                         <  1:237366/301‑1 (MQ=255)`  
                                                                                                                                                                                                                                   `tctcGCAGTGACCCAGAAGGGTGACGCATGGATTCGTGCGTTACAGGATACTATTCGTGAACTGCAACGTAAGCAGGAAATCAAGTAAGGAGGCAATGTGTCTACTCAATCCAATCGTAATGCGCTCGTAGTGGCGCAACTGAAAGGAGACTTCGTGGCGTTCCTATTCGTCTTATGGAAGGCGCTAAACCTACCGGTGCCCACTAAGTGTCAGATTGACATGGCTAAGGTGCTGGCGAATGGAGACAACAAGAAGTTCATCTTACAGGCTTTCCGTGGTATCGGTAAGTCGTTCATcaca                                                                        <  1:244309/301‑1 (MQ=255)`  
                                                                                                                                                                                                                                             `aCCCAGAAGGGTGACGCATGGATTCGTGCGTTACAGGATACTATTCGTGAACTGCAACGTAAGCAGGAAATCAAGTAAGGAGGCAATGTGTCTACTCAATCCAATCGTAATGCGCTCGTAGTGGCGCAACTGAAAGGAGACTTCGTGGCGTTCCTATTCGTCTTATGGAAGGCGCTAAACCTACCGGTGCCCACTAAGTGTCAGATTGACATGGCTAAGGTGCTGGCGAATGGAGACAACAAGAAGTTCATCTTACAGGCTTTCCGTGGTATCGGTAAGTCGTTCATCACATGttcgttcg                                                              >  1:43783/1‑301 (MQ=255)`  
                                                                                                                                                                                                                                              `cccAGAAGGGTGACGCATGGATTCGTGCGTTACAGGATACTATTCGTGAACTGCAACGTAAGCAGGAAATCAAGTAAGGAGGCAATGTGTCTACTCAATCCAATCGTAATGCGCTCGTAGTGGCGCAACTGAAAGGAGACTTCGTGGCGTTCCTATTCGTCTTATGGAAGGCGCTAAACCTACCGGTGCCCACTAAGTGTCAGATCGACATGGCTAAGGTGCTGGAGAATGGAGACAACAAGAAGTTCATCTTACAGGCTTTCCGTGGTATCGGTAAGTCGTTTATCACATGTGcgttcgt                                                             >  1:124290/1‑301 (MQ=255)`  
                                                                                                                                                                                                                                                  `gAAGGGTGACGCATGGATTCGTGCGTTACAGGATACTATTCGTGAACTGCAACGTAAGCAGGAAATCAAGTAAGGAGGCAATGTGTCTACTCAATCCAATCGTAATGCGCTCGTAGTGGCGCAACTGAAAGGAGACTTCGTGGCGTTCCTATTCGTCTTATGGAAGGCGCTAAACCTACCGGTGCCCACTAAGTGTCAGATTGACATGGCTAAGGTGCTGGCGAATGGAGACAACAAGAAGTTCATCTTACAGGCTTTCCGTGGTATCGGTAAGTCGTTCATCACATGTGCGTTCGTtgtg                                                         <  1:16387/301‑1 (MQ=255)`  
                                                                                                                                                                                                                                                     `gggTGACGCATGGATTCGTGCGTTACAGGATACTATTCGTGAACTGCAACGTAAGCAGGAAATCAAGTAAGGAGGCAATGTGTCTACTCAATCCAATCGTAATGCGCTCGTAGTGGCGCAACTGAAAGGAGACTTCGTGGCGTTCCTATTCGTCTTATGGAAGGCGCTAAACCTACCGTTGCCCACTAAGTGTCAGATTGACATGGCTAAGGTGCTGGCGAATGGAGACAACAAGAAGTTCATCTTACAGGCTGTCCGTGGTATCGGTAAGACGTTCATCACATGTGCGTTAGTTGTGTgg                                                      >  1:61552/1‑301 (MQ=255)`  
                                                                                                                                                                                                                                                        `taaCGCATGGATTCGTGCGTTACAGGATACTATTCGTGAACTGCAACGTAAGCAGGAAATCAAGTAAGGAGGCAATGTGTCTACTCAATCCAATCGTAATGCGCTCGTAGAGGCGCAACTGAAAGGAGACTTCGTGGCGTTCCTATTCGTCTTATGGAAGGCGCTAAACCTACCGGTGCCCACTAAGTGTCAGATTGACATGACTAAGGTGCAGGCGAATGGAGACAACAAGAAGTTCATCTTACAGGCTTTCCGTGGTATCGGTAAGTCGTTCATCACATGTGCGTTCGTTGTGTGGTcc                                                   <  1:234371/299‑1 (MQ=255)`  
                                                                                                                                                                                                                                                        `tGACGCATGGATTCGTGCGTTACAGGATACTATTCGTGAACTGCAACGTAAGCAGGAAATCAAGTAAGGAGGCAATGTGTCTACTCAATCCAATCGTAATGCGCTCGTAGTGGCGCAACTGAAAGGAGACTTCGTGGCGTTCCTATTCGTCTTATGGAAGGCGCTAAACCTACCGGTGCCCACTAAGTGTCAGATTGACATGGCTAAGGTGCTGGCGAATGGAGACAACAAGAAGTTCATCTTACAGGCTTTCCGTGGTATCGGTAAGTCGTTCATCACATGTGCGTTCGTTGTGTGGTcc                                                   <  1:241333/301‑1 (MQ=255)`  
                                                                                                                                                                                                                                                        `ggACGCATGGATTCGTGCGTTACAGGATACTATTCGTGAACTGCAACGTAAGCAGGAAATCAAGTAAGGAGGCAATGTGTCTACTCAATCCAATCGTAATGCGCTCGTAGTGGCGCAACTGAAAGGAGACTTCGTGGCGTTCCTATTCGTCTTATGGAAGGCGCTAAACCTACCGGTGCCCACTAAGTGTCAGATTGACATGGCTAAGGTGCTGGCGAATGGAGACAACAAGAAGTTCATCTTACAGGCTTTCCGTGGTATCGGTAAGTCGTTCATCACATGTGCGTTCGTTGTGTGGTcc                                                   <  1:116996/300‑1 (MQ=255)`  
                                                                                                                                                                                                                                                           `cGCATGGATTCGTGCGTTACAGGATACTATTCGTGAACTGCAACGTAAGCAGGAAATCAAGTAAGGAGGCAATGTGTCTACTCAATCCAATCGTAATGCGCTCGTAGTGGCGCAACTGAAAGGAGACTTCGTGGCGTTCCTATTCGTCTTATGGAAGGCGCTAAACCTACCGGTGCCCACTAAGTGTCCGATTGACATGGCTAAGGTGTTGCCGAATGGAGCCAACAAGAAGTTCATCTTACAGGCTTTCCGTGGTATCGGTAAGTCGTTCATCTCATTTGCGTTCGTTGTGTGGTCCttt                                                >  1:230641/1‑300 (MQ=255)`  
                                                                                                                                                                                                                                                           `cGCATGGATTCGTGCGTTACAGGATACTATTCGTGAACTGCAACGTAAGCAGGAAATCAAGTAAGGAGGCAATGTGTCTACTCAATCCAATCGTAATGCGCTCGTAGTGGCGCAACTGAAAGGAGACTTCGTGGCGTTCCTATTCGTCTTATGGAAGGCGCTAAACCTACCGGTGCCCACTAAGTGTCAGATTGACATGGCTAAGGTGCTGGCGAATGGAGACAACAAGAAGTTCATCTTACAGGCTTTCCGTGGTATCGGTAAGTCGTTCATCACATGTTCGTTCGTTGTGTGGTCCTTa                                                >  1:51357/1‑301 (MQ=255)`  
                                                                                                                                                                                                                                                           `cGCATGGATTCGTGCGTTACAGGATACTATTCGTGAACTGCAACGTAAGCAGGAAATCAAGTAAGGAGGCAATGTGTCTACTCAATCCAATCGTAATGCGCTCGTAGTGGCGCAACTGAAAGGAGACTTCGTGGCGTTCCTATTCGTCTTATGGAAGGCGCTAAACCTACCGGTGCCCACTAAGTGTCAGATTGACATGGCTAAGGTGCTGGCGAATGGAGACAACAAGAAGTTCATCTTACAGGCTTTCCGTGGTATCGGTAAGTCGTTCATCACATGTGCGTTCGTTGTGTGGTCCttt                                                >  1:67521/1‑300 (MQ=255)`  
                                                                                                                                                                                                                                                            `gCATGGTTTCGTTCGTTACAGGATACTATTCGTGAACTGCAACGTAAGCAGGAAATCAAGTAAGGAGGCAATGTGTCTACTCAATCCAATCGTAATGCGCTCGTAGTGGCGCAACTGAAAGGAGACTTCGTGGCGTTCCTATTCGTCTTATGGAAGGCGCTAAACCTACCGGTGCCCACTAAGTGTCAGATTGACATGGCTAAGGTGCTGGCGAATGGAGACAACAAGAAGTTCATCTTACAGGCTTTCCGTGGTATCGGTAAGTCGTTCATCACATGTGCGTTCGTTGTGTGGTCCTTAt                                               <  1:332451/301‑1 (MQ=255)`  
                                                                                                                                                                                                                                                              `gtgtataagagacagaCAGGATACTATTCGTGAACTGCAACGTAAGCAGGAAATCAAGTAAGGAGGCAATGTGTCTACTCAATCCAATCGTAATGCGCTCGTAGTGGCGCAACTGAAAGGAGACTTCGTGGCGTTCCTATTCGTCTTATGGAAGGCGCTAAACCTACCGGTGCCCACTAAGTGTCAGATTGACATGGCTAAGGTGCTGGCGAATGGAGACAACAAGAAGTTCATCTTACAGGCTTTCCGTGGTATCGGTAAGTCGTTCATCACATGTGCGTTCGTTGTGTGGTCCTTATgg                                             <  1:35050/286‑1 (MQ=255)`  
                                                                                                                                                                                                                                                                `ggATTCGTGCGTTACAGGATACTATTCGTGAACTGCAACGTAAGCAGGAAATCAAGTAAGGAGGCAATGTGTCTACTCAATCCAATCGTAAGGCGCTCGTAGTGGCGCAACTGAAAGGAGACTTCGTGGCGTTCCTATTCGTCTTATGGAAGGCGCTAAACCTACCGGTGCCCACTAAGTGTCAGATTGACATGGCTAAGGTGCTGGCGAATGGAGACAACAAGAAGTTCATCTTACAGGCTTTCCGTGGTATCGGTAAGTCGTTCATCACATGTGCGTTCGTTGTGTGGTCCTTATGgag                                           <  1:81993/301‑1 (MQ=255)`  
                                                                                                                                                                                                                                                                `ggATTCCTGCGTTACAGGAGACCATTCGTGAACTGCAACGTAAGCAGGAAATCAAGTAAGGAGGCAATGTGTCTACTCAATCCAATCGTAATGCGCTCGTAGTGGCGCAACTGAAAGGAGACTTCGTGGCGTTCCTATTCGTCTTATGGAAGGCGCTAAACCTACCGGTGCCCACTAAGTGTCAGATTGACATGGCTAAGGTGCTGGCGAATGGAGACAACAAGAAGTTCATCTTACAGGCTTTCCGTGGTATCGGTAAGTCGTTCATCACATGTGCGTTCGTTGTGTGGTCCTTATGgag                                           <  1:338518/301‑1 (MQ=255)`  
                                                                                                                                                                                                                                                                     `agagacagaCAGGATACTATTCGTGAACTGCAACGTAAGCAGGAAATCAAGTAAGGAGGCAATGTGTCTACTCAATCCAATCGTAATGCGCTCGTAGTGGCGCAACTGAAAGGAGACTTCGTGGCGTTCCTATTCGTCTTATGGAAGGCGCTAAACCTACCGGTGCCCACTAAGTGTCAGATTGACATGGCTAAGGTGCTGGCGAAAGGAGACAACAAGAAGTTCATCTTACAGGCTTTCCGTTGTATCGGTAAGTCGTTCATCACATGTGCGTTCGTTGTGTGGTCCTTATGGAGAGAcc                                      <  1:138773/293‑1 (MQ=255)`  
                                                                                                                                                                                                                                                                           `ttACAGGATACTATTCGTGAACTGCAACGTAAGCAGGAAATCAAGTAAGGAGGCAATGTGTCTACTCAATCCAATCGTAATGCGCTCGTAGTGGCGCAACTGAAAGGAGACTTCGTGGCGTTCCTATTCGTCTTATGGAAGGCGCTAAACCTACCGGTGCCCACTAAGTGTCAGATTGACATGGCTAAGGTGCTGGCGAATGGAGACAACAAGAAGTTCATCTTACAGGCTTTCCGTGGTATCGGTAAGTCGTTCATCACATGTGCGTTCGTTGTGTGGTCCTTATGGAGAGACCCTCAGt                                >  1:135725/1‑301 (MQ=255)`  
                                                                                                                                                                                                                                                                           `ttACAAGATACTATTCGTGAAATGGAACGTAAGGAGGAAATCAAGTAAGGAGGCAATGTGTCTACTCAATCCAATCGTAATGCGCTCGTAGTGGCGCAACTGAAAGGAGACTTCGTGGCGTTCCTATTCGTCTTATGGAAGGCGCTAAACCTACCGGTGCCCACTAAGTGTCAGATTGACATGGCTAAGGTGCTGGCGAATGGAGACAACAAGAAGTTCATCTTACAGGATTTCCGTGGTATCGGTAAGTCGTTCATCACATGTGCGTTCGTTGTGTGGTCCTTATGGAGAGACACTCAGt                                <  1:322959/301‑1 (MQ=255)`  
                                                                                                                                                                                                                                                                             `aCAGGATACTATTCGTGAACTGCAACGTAAGCAGGAAATCAAGTAAGGAGGCAATGTGTCTACTCAATCCAATCGTAATGCGCTCGTAGTGGCGCAACTGAAAGGAGACTTCGTGGCGTTCCTATTCGTCTTATGGAAGGCGCTAAACCTACCGGTGCACACTCAGTGTCAGATTGACATGGCTAAGGTGCTGGCGAATGGAGACAACAAGAAGTTCATCTTACAGGCTTTCCGTGGTATCGGTAAGTCGTTCATCACATGTGCGTTCGTTGTGTGGTCCTTATGGAGTGACCCTCAGTTg                              >  1:274856/1‑301 (MQ=255)`  
                                                                                                                                                                                                                                                                                `ggATACTATTCGTGAACTGCAACGTAAGCAGGAAATCAAGTAAGGAGGCAATGTGTCTACTCAATCCAATCGTAATGCGCTCGTAGTGGCGCACCTGAAAGGAGACTTCGTGGCGTTCCTATTCGTCTTATGGAAGGCGCTAAACCTACCGGTGCCCACTAAGTGTCAGATTGACATGGCTAAGGTGCTGGCGAATGGAGACAACAAGAAGTTCATCTTACAGGCTTTCCGTGGTATCGGTAAGTCGTTCATCACATGTGCGTTCGTTGTGTGGTCCTTATGGAGAGACCCTCAGTTGAAg                           >  1:28689/1‑301 (MQ=255)`  
                                                                                                                                                                                                                                                                                `ggATACTATTCGTGAACTGCAACGTAAGCAGGAAATCAAGTAAGGAGGCAATGTGTCTACTCAATCCAATCGTAATGCGCTCGTAGTGGCGCAACTGAAAGGAGACTTCGTGGCGTTCCTATTCGTCTTATGGAAGGCGCTAAACCTACCGGTGCCCACTAAGTGTCAGATTGACATGGCTAAGGTGCTGGCGAATGGAGACAACAAGAAGTTCATCTTACAGGCTTTCCGTGGTATCGGTAAGTCGTTCATCACATGTGCGTTCGTTGTGTGGTCCTTATGGAGAGACCCTCAGTTGAAg                           >  1:169181/1‑301 (MQ=255)`  
                                                                                                                                                                                                                                                                                `ggATACTATTCGTGAACTGCAACGTAAGCAGGAAATCAAGTAAGGAGGCAATGTGTCTACTCAATCAAATCGTAATGCGCTCGTAGTGGCGCAACTGAAAGGAGACTTCGTGGCGTTCCTATTCGTCTTATGGAAGGCGCTAAACCTACCGGTGCCCACTAAGTGTCAGATTGACATGGCTAAGGTGCTGGCTACTGGATACAACAAGAAGTTCATCTTACAGGCTTTCCGTGGTATCGGTAAGTCGTTCATCACATGTGCGTTCGTTGTGTGGTCCTTATGGAGTGACCCTCAGTTGAAg                           >  1:19957/1‑301 (MQ=255)`  
                                                                                                                                                                                                                                                                                  `aagagacaggTGAACTGCAACGTAAGCAGGAAATCAAGTAAGGAGGGAATGTGTCTACTCAATCCAATCGTAATGCGCACGTAGTGGCGCAACTGAAAGGAGACTTCGTGGCGTTCCTATTCGTCTTATGGAAGGCGCTAAACCTACCGGTGCCCACTAAGTGTCAGATTGACATGGCTAAGGTGCTGGCGAATGGAGACAACAAGAAGTTCATCTTACAGGCTTTCCGTGGTATCGGTAAGTCGTTCATCACATGTGCGTTCGTTGTGTGGTCATTATGGAGAGACCCTCAGTTGAAGAt                         <  1:114045/292‑1 (MQ=255)`  
                                                                                                                                                                                                                                                                                  `aTACTATTCGTAAACTGCAACGTAAGCAGGAAAGCAAGTAAGGAGGCAATGTGTCTACTCAATCCAATCGTAATGCGCTCGTAGTGGCGCAACTGAAAGGAGACTTCGTGGCGTTCCTATTCGTCTTATGGAAGGCGCTAAACCTACCGGTGCCCACTAAGTGTCAGATTGACATGGCTAAGGTGCTGGCGAATGGAGACAACAAGAAGTTCATCTTACAGGCTTTCCGTGGTATCGGTAAGTCGTTCATCACATGTGCGTTCGTTGTGTGGTCCTTATGGAGAGACCCTCAGTTGAAGAt                         <  1:39405/301‑1 (MQ=255)`  
                                                                                                                                                                                                                                                                                     `cTATTCGTGAACTGCAACGTAAGCAGGAAATCAAGTAAGGAGGCAATGTGTCTACTCAATCCAATCGTAATGCGCTCGTAGTGGCGCAACTGAAAGGAGACTTCGTGGCGTTCCTATTCGTCTTATGGAAGGCGCTAAACCTACCGGTGCCCACTAAGTGTCAGATTGACATGGCTAAGGTGCTGGCGAATGGAGACAACAAGAAGTTCATCTTACATGCTTTCCGTGGTATCGGTAAGTCGTTCATCACATGTTCGTTCGTTGTGTGGTCCTTATGGAGAGACCCTCTGTTGAAGATACt                      >  1:132193/1‑301 (MQ=255)`  
                                                                                                                                                                                                                                                                                        `ttCGTGAACTGCAACGTAAGCAGGAAATCAAGTAAGGAGGCAATGTGTCTACTCAATCCAATCGTAATGCGCTCGTAGTGGCGCAACTGAAAGGAGACTTCGTGGCGTTCCTATTCGTCTTATGGACGGCGCTAAACCTACCGGTGCCCACTAAGTGTCAGATTGACATGTCTAAGGTGCTGGCGAATGGAGACAACAAGAAGTTCATCTTACAGGCTTTCCGTGGTATCGGTAAGTCGTTCATCACCTGTGCGTTCGTTGTGTGGTCCTTATGGTGAGACCCTCAGTTGAAGATACTTAt                   >  1:227243/1‑301 (MQ=255)`  
                                                                                                                                                                                                                                                                                          `cGTGAACTGCAACGTAAGCAGGAAATCAAGTAAGGAGGCAATGTGTCTACTCAATCCAATCGAAATGCGCTCGTAGTGGCGCAACTGAAAGGAGACTTCGTGGCGTTCCTATTCGTCTTATGGAAGGCGCTAAACCTACCGGTGCCCACTAAGTGTCAGATTGACATGGCTAAGGTGCTGGCGAATGTAGACAACAAGAAGTTCATCTTACAGGCTTTCCGTGGTATCGGTAAGTCGTTCATCACATGTGCGTTCGTTGTGTGGTCCTTATGGAGAGACCCTCAGTTGAAGATACTTATCg                 <  1:78216/301‑1 (MQ=255)`  
                                                                                                                                                                                                                                                                                            `taaaCTGCAACGTAAGCAGGAAATCAAGTAAGGAGGCAATGTGTCTACTCAATCCAATCGTAATGCGCTCGTAGTGGCGCAACTGAAAGGAGACTTCGTGGCGTTCCTATTCGTCTTATGGAAGGCGCTAAACCTACCGGTGCCCACTAAGTGTCAGATTGACATGGCTAAGGTGCTGGCGAATGGAGACAACAAGAAGTTCATCTTACAGGCTTTCCGTGGTATCGGTAAGTCGTTCATCACATGTGCGTTCGTTGTGTGGTCCTTATGGAGAGACCCTCAGTTGAAGATACTTATCGTa               <  1:152304/299‑1 (MQ=255)`  
                                                                                                                                                                                                                                                                                              `aaCTGCAACGTAAGCAGGAAATCAAGTAAGGAGGCAATGTGTCTACTCAATCCAAGCGTAATGCGCTCGTAGTGGCGCAACTGAAAGGAGACGTCGTGGCGTTACTATTCGTCTTATGGAAGGCGCTAAACCTACCGGTGCACACTAAGTGTCAGATTGACATGGCTAAGGTGCTGGCGAATGGAGACAACAAGAAGTTCATCTTACAGGCTTTCCGTGGTATCGGTAAGTCGTTCATCACATGTGCGTTCGTTGTGTGGTCCTTATGGAGAGACCCTCAGTTGAAGATACTTATCGTATc             <  1:336206/301‑1 (MQ=255)`  
                                                                                                                                                                                                                                                                                              `aaCTGCAACGTAAGAAGGAAATAAAGTAAGGAGGCAATGTGTCTACTCAATCCAATCGTAATGCGCGCGTAGTGGCGCAACTGAAAGGAGACTTCGTGGCGTTCCTATTCGTCTTATGGAAGGCGCTAAACCTACCGGTGCCCACTAAGTGTCAGATTGACATGGCTAAGGTGCTGGCGAATGGAGACAACAAGAAGTTCATCTTACAGGCTTTCCGTGGTATCGGTAAGTCGTTCATCACATGTGCGTTCGTTGTGTGGTCCTTATGGAGAGACCCTCAGTTGAAGATACTTATCGTATc             <  1:31690/301‑1 (MQ=255)`  
                                                                                                                                                                                                                                                                                                  `gCAACGTAAGCAGGAAATCAAGTAAGGAGGCAATGTGTCTACTCAATCCAATCGTAATGCGCTCGTAGTGGCGCAACTGAAAGGAGACTTCGTGGCGTTCCTATTCGTCTTATGGAAGGCGCTAAACCTACCGGTGCCCACTAAGTGTCAGATTGACATGGCTAAGGTGCTGGCGAATGGAGACAACAAGAAGTTCATCTTACAGGCTTTCCGTGGTATCGGTAAGTCGTTCATCACATGTGCGTTCGTTGTGTGGTCCTTATGGAGAGACCCTCAGTTGAAGATACTTATCGTATCAGcc         <  1:258423/301‑1 (MQ=255)`  
                                                                                                                                                                                                                                                                                                  `ccAACGTAAGCAGGAAATCAAGTAAGGAGGCAATGTGTCTACTCAATCCAATCGTAATGCGCTCGTAGTGGCGCAACTGAAAGGAGACTTCGTGGCGTTCCTATTCGTCTTATGGAAGGCTCTAAACCGACCGGTGCCCACTAAGTGTCAGATTGACATGGCTAAGGTGCTGGCGAATGGAGACAACAAGAAGTTCATCTTACAGGCTTTCCGTGGTATCGGTAAGTCGTTCATCACATGTGCGTTCGTTGTGTGGTCCTTATGGAGAGACCCTCAGTTGAAGATACTTATCGTATCAGcc         <  1:167319/300‑1 (MQ=255)`  

                                                                                                                                                                                                                                                                                                            `|`                                                                                                                                                                                                                                                                                                           
`AGAGTTGAGGCTGCGAAGAGCACTCAAAGAGCAATCGATGCGGTATCTGCTAAGTATCAAGAAGACCTTGCCGCGCTGGAAGGGAGCACTGATAGGATTATTTCTGATTTGCGTAGCGACAATAAGCGGTTGCGCGTCAGAGTCAAAACTACCGGAACCTCCGATGGTCAGTGTGGATTCGAGCCTGATGGTCGAGCCGAACTTGACGACCGAGATGCTAAACGTATTCTCGCAGTGACCCAGAAGGGTGACGCATGGATTCGTGCGTTACAGGATACTATTCGTGAACTGCAACGTAAGTAGGAAATCAAGTAAGGAGGCAATGTGTCTACTCAATCCAATCGTAATGCGCTCGTAGTGGCGCAACTGAAAGGAGACTTCGTGGCGTTCCTATTCGTCTTATGGAAGGCGCTAAACCTACCGGTGCCCACTAAGTGTCAGATTGACATGGCTAAGGTGCTGGCGAATGGAGACAACAAGAAGTTCATCTTACAGGCTTTCCGTGGTATCGGTAAGTCGTTCATCACATGTGCGTTCGTTGTGTGGTCCTTATGGAGAGACCCTCAGTTGAAGATACTTATCGTATCAGCCTCTAAGG  >  NC_001604.1/37046‑37643`

  

Alignment Legend

Aligned base mismatch/match (shaded by quality score): `ATCG/ATCG` `< 3 ≤` `ATCG/ATCG` `< 11 ≤` `ATCG/ATCG` `< 24 ≤` `ATCG/ATCG` `< 36 ≤` `ATCG/ATCG` `< 38 ≤` `ATCG/ATCG`

Unaligned base: `atcg`    Masked matching base: `atcg`    Alignment gap: `‑`    Deleted base: `‑`
